# MAGs-based genomic comparison of gut significantly enriched microbes in obese individuals pre- and post-bariatric surgery across diverse locations

**DOI:** 10.3389/fcimb.2025.1485048

**Published:** 2025-03-18

**Authors:** Hang Shi, Jia Li

**Affiliations:** Plastic Surgery Hospital, Chinese Academy of Medical Sciences and Peking Union Medical College, Beijing, China

**Keywords:** obesity, bariatric surgeries, gut microbiota, MAGs, fatty acid biosynthesis

## Abstract

**Introduction:**

Obesity, a pressing global health issue, is intricately associated with distinct gut microbiota profiles. Bariatric surgeries, such as Laparoscopic Sleeve Gastrectomy (LSG), Sleeve Gastrectomy (SG), and Roux-en-Y Gastric Bypass (RYGB), induce substantial weight loss and reshape gut microbiota composition and functionality, yet their comparative impacts remain underexplored.

**Methods:**

This study integrated four published metagenomic datasets, encompassing 500 samples, and employed a unified bioinformatics workflow for analysis. We assessed gut microbiota α-diversity, identified species biomarkers using three differential analysis approaches, and constructed high-quality Metagenome-Assembled Genomes (MAGs). Comparative genomic, functional profiling and KEGG pathway analyses were performed, alongside estimation of microbial growth rates via Peak-to-Trough Ratios (PTRs).

**Results:**

RYGB exhibited the most pronounced enhancement of gut microbiota α-diversity compared to LSG and SG. Cross-cohort analysis identified 39 species biomarkers: 27 enriched in the non-obesity group (NonOB_Enrich) and 12 in the obesity group (OB_Enrich). Among the MAGs, 177 were NonOB_Enrich and 14 were OB_Enrich. NonOB_Enrich MAGs displayed enriched carbohydrate degradation profiles (e.g., GH105, GH2, GH23, GH43, and GT0 families) and higher gene diversity in fatty acid biosynthesis and secondary metabolite pathways, alongside significant enrichment in amino acid metabolism (KEGG analysis). Post-surgery, Akkermansia muciniphila and Bacteroides uniformis showed elevated growth rates based on PTRs.

**Discussion:**

These findings underscore RYGB’s superior impact on gut microbiota diversity and highlight distinct microbial functional adaptations linked to weight loss, offering insights for targeted therapeutic strategies.

## 1 Introduction

The gut microbiota is essential for human health, impacting metabolism, immune function, and overall well-being (Clemente et al., 2012). Obesity, a widespread global health concern, is linked to distinct gut microbiota profiles (Turnbaugh et al., 2006). Bariatric surgeries, such as Laparoscopic Sleeve Gastrectomy (LSG), Sleeve Gastrectomy (SG), and Roux-en-Y gastric bypass (RYGB), are highly effective treatments for severe obesity, resulting in significant weight loss and metabolic improvements (Zhang et al., 2009). These procedures also lead to notable changes in the composition and function of the gut microbiota (Aron-Wisnewsky et al., 2019; Lee et al., 2019). Additionally, identifying novel weight loss-related microbes and understanding their mechanisms of action are crucial for advancing human health and the development of the health industry (O’Toole et al., 2017). This study integrates data from multi-cohort data to identify weight loss-related microbe biomarkers.

Variations in study designs, experimental procedures, and bioinformatics workflows have contributed to inconsistencies in research findings. For example, studies have reported conflicting outcomes regarding the relative abundance of the phylum Bacteroidetes and the populations of Faecalibacterium and Bifidobacterium species following bariatric surgery, with some indicating an increase and others a decrease (Chen et al., 2017; Fouladi et al., 2021; Furet et al., 2010; Murphy et al., 2017). Furthermore, some studies investigating the impact of RYGB on gut microbiota (Palleja et al., 2016) have used the Wilcoxon test to identify differential species, which may result in unacceptably high false positive rates (Nearing et al., 2022). In this study, by employing three differential analysis methods—LEfSe, ANCOM, and edgeR—we identified consistently significant differential species across cohorts. This highlights the necessity of integrating multiple methods for a comprehensive analysis to identify more reliable species-level biomarkers related to obesity or weight loss. Additionally, there is currently no comparative study examining the impact of different surgical methods on gut microbiota diversity. In this study, we compared the effects of gut microbiota recovery under different surgical methods using a unified and standardized bioinformatics analysis workflow.

As the study (Fouladi et al., 2021) analyzing multi-cohort data to identify replicable post-RYGB gut microbiota and metabolic pathway shifts (e.g., increased *Veillonella*/*Akkermansia* and decreased *Blautia*) conducted a meta-analysis mainly based on existing 16S rRNA research, there is a notable lack of meta-analyses at the metagenomic level. This study addresses this gap by utilizing four published metagenomic datasets, encompassing 500 samples related to bariatric surgery, and applying a unified bioinformatics analysis workflow. Metagenome-Assembled Genomes (MAGs) construction, a culturing-independent and reference-free approach, offers a promising strategy for uncovering microbial diversity and genomic features (Saheb Kashaf et al., 2022; Zeng et al., 2022). This study aims to address this gap by using MAGs to conduct a comparative genomic analysis of NonOB_Enrich MAGs and OB_Enrich MAGs across diverse geographical locations to elucidate the potential genetic mechanisms underlying weight gain or loss.

This study highlights the importance of metagenomic approaches in understanding the complex interplay between gut microbiota and obesity, offering potential avenues for optimizing bariatric surgery and enhancing patient health (Qin et al., 2012). By elucidating the microbial shifts associated with bariatric surgery, this research offers valuable insights for developing targeted probiotic therapies to enhance obesity treatment.

## 2 Methods

### 2.1 Datasets in this study

This study comprises four distinct cohorts designed to analyze the metagenomic profiles of gut microbiota in obese individuals before and after bariatric surgery across different geographical locations. The study population undergoing weight loss interventions excluded individuals with basal metabolic diseases, cancer, and other related conditions. The samples were derived from consistent tissue types and uniform sequencing methods were employed. After screening, a total of four cohorts were included in the study (**Table 1**), as detailed below:

**Table 1:** The four distinct cohorts designed to analyze the metagenomic profiles of gut microbiota in obese individuals before and after bariatric surgery across different geographical locations in this study.

Project	Study title	Sequence	Treatment	Sample type	Position	Total of samples	Healthy	Obesity (OB)	SG pre	SG 1M	SG 3M	RYGB pre (OB)	RYGB 1M	RYGB 3M	RYGB 6M	RYGB 12M	LSG pre (OB)	LSG 6M
PRJEB12123	Gut microbiome and serum metabolome alterations in obesity and after weight-loss intervention	PE 100bp	SG	faeces	China:Shanghai	256	105	88	23	17	23	–	–	–	–	–	–	–
PRJNA597839	A metagenome-wide association study of gut microbiome and visceral fat accumulation	PE 150bp	LSG	faeces	China:Shanghai	76	30	–	–	–	–	–	–	–	–	–	32	14
																			PRJEB12947	Roux-en-Y gastric bypass surgery of morbidly obese patients induces swift and persistent changes of the individual gut microbiota	PE 100bp	RYGB	faeces	Denmark	33	–	–	–	–	–	13	–	12	–	8	–	–
PRJNA668357	A microbial signature following bariatric surgery is robustly consistent across multiple cohorts	PE 150bp	RYGB	faeces	USA	135	–	52	–	–	–	–	38	–	27	18	–	–

Cohort 1 - PRJEB12123 (Liu et al., 2017). This cohort includes 256 fecal samples collected from Beijing, China. The samples include 105 healthy individuals, 88 obese individuals, and 63 individuals at various stages post-Sleeve Gastrectomy (SG). Specifically, the post-SG samples are categorized into SG 0M (23 samples), SG 1M (17 samples), and SG 3M (23 samples). This cohort uses paired-end sequencing with a read length of 100bp.

Cohort 2 - PRJNA597839 (Nie et al., 2020): Comprising 76 fecal samples from the USA, this cohort focuses on the longitudinal analysis of patients undergoing laparoscopic Sleeve Gastrectomy (LSG). It includes 30 healthy individuals and 46 obese individuals pre- and post-surgery. The samples collected include LSG pre (32 samples) and LSG 6M (14 samples). Sequencing is performed with paired-end reads of 150bp.

Cohort 3 - PRJEB12947 (Palleja et al., 2016): This cohort consists of 33 fecal samples collected from Beijing, China, specifically focusing on patients undergoing Roux-en-Y gastric bypass (RYGB). This cohort focuses on longitudinal samples from patients undergoing RYGB, without healthy controls: RYGB pre (13 samples), RYGB 1M (1 sample), RYGB 3M (12 samples), and RYGB 12M (8 samples). Paired-end sequencing with 2x100bp reads is utilized for this cohort.

Cohort 4 - PRJNA668357 (Fouladi et al., 2021): The final cohort involves 135 fecal samples from the USA, targeting the analysis of gut microbiota in patients undergoing RYGB surgery. This cohort includes 52 obese individuals and post-surgery samples: RYGB pre (38 samples), RYGB 1M (0 samples), RYGB 3M (27 samples), and RYGB 12M (18 samples). The sequencing for this cohort is performed with paired-end reads of 2x150bp.

### 2.2 Sequence data acquisition and preprocessing

Sequence read archive (SRA) files of all samples were downloaded using prefetch software. Raw sequence data were subjected to quality control using fastp (https://github.com/OpenGene/fastp). Human-derived sequences were removed by aligning the reads to the human genome using Bowtie2 (v2.3.4.3). Next, these non-human sequences were aligned to a reference database with Kraken2 (Wood et al., 2019). As a reference database, we used the Kraken-build utility to download bacterial, archaeal, viral and fungal libraries including National Centre for Biotechnology Information (NCBI) taxonomic information as well as complete genome sequences from RefSeq (Jing et al., 2021; Nearing et al., 2022; Sola-Leyva et al., 2021). In addition, we also incorporated the human and protozoa genome into the Kraken2 database to further reduce false-positive microbes introduced by human-derived or other eukaryotic sequences. Based on the effective clean data volume from the sequencing of each sample, we used the RPTM (Reads Per Ten Million) normalization method to standardize the data at the species level, followed by subsequent diversity and differential species analyses. Besides, the filtered reads were then assembled denovo using megahit (https://github.com/voutcn/megahit). Metagenome-Assembled Genomes (MAGs) were constructed using metaWRAP (https://github.com/bxlab/metaWRAP). The bin refinement module in metaWRAP was employed to optimize MAG quality, followed by dereplication using dRep (https://github.com/MrOlm/drep,v3.2.2) to remove redundant MAGs. Taxonomic classification of the MAGs was performed using the classify-wf module in GTDB-Tk. Genomic k-mer distances among MAGs were calculated using mash (v2.3, https://mash.readthedocs.io/en/latest/). A phylogenetic tree was constructed with the Neighbor-Joining method (nj) using the ape package in R. The resulting tree was visualized using the Interactive Tree Of Life (iTOL) web tool. Genes within the MAGs were predicted by prodigal (v2.6.3) and annotated by aligning them to the CAZy and KEGG databases using the blastp module of DIAMOND (https://github.com/bbuchfink/diamond). Functional genes were identified and their abundances were quantified for each MAG. CoPTR (v1.1.6) was used to compute peak-to-trough ratios (PTRs) from MAGs to accurately reflect microbial growth rates. Gapseq (v1.3.1) was used to predict metabolic processes (Zimmermann et al., 2021). Prediction of secondary metabolites in each MAG was performed using antiSMASH (v7.0.1).

### 2.3 Statistical analysis

The effects of different cohorts, sample locations, and experimental groups on microbial community composition were assessed using the PERMANOVA test in the vegan package of R (p-value < 0.05 was considered significant). Differential abundance testing to identify differentially abundant species between obese and healthy individuals (OB-Healthy) and between obese individuals pre- and post-treatment (OB-Treat) employed three methods: edgeR v3.36.0 (FDR < 0.05, FC = 5), ANCOM v2.1 (detection threshold 0.9), and LEfSe v1.0 (logarithmic LDA score > 2). Core differential species were identified based on consensus results from these methods across multiple cohorts, ensuring robust identification of key species affected by obesity and treatment. The Kruskal-Wallis test was used to compare PTR differences between groups. Differential KOs were subjected to KEGG enrichment analysis using R clusterProfiler. We calculated the within-sample α-diversity using Shannon and ACE index on species level to estimate the richness of samples using QIIME (Caporaso et al., 2010) v1.9.1. Besides, the post-surgery α-diversity index increased ratio refers to the proportional increase in α-diversity index after surgery compared to preoperative levels. Specifically, we calculated this ratio by: (postoperative α-diversity index - preoperative α-diversity index)/preoperative α-diversity index for each cohort. Variance partitioning analysis was performed by varpart function of vegan packages.

## 3 Result

### 3.1 Cohort, location, and health factors significantly affect gut microbiota diversity

**Figure 1:**
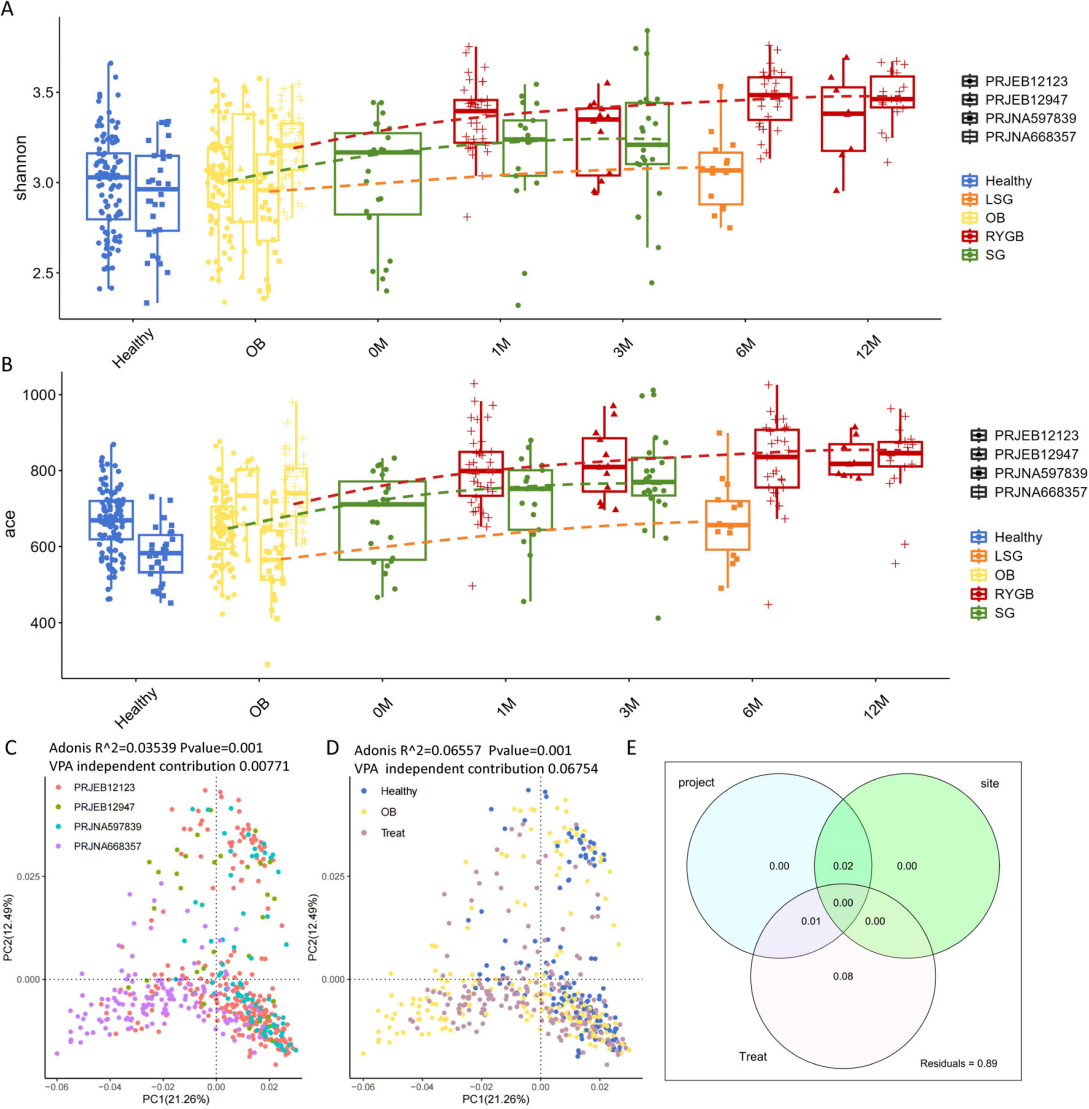
Microbial community diversity comparison. **(A)** shannon index and **(B)** ace index comparison of different groups for each cohort. **(C)** PCoA plot showing significant differences between the four cohorts (PRJEB12123, PRJEB12947, PRJNA597839, and PRJNA668357) **(D)** PCoA plot presenting the distribution of microbial communities among OB, health and treat status. The curves represent the trend lines of the diversity index changes for each surgical method. **(E)** Venn diagram showing variance partitioning analysis (VPA) for project, site, and treatment factors. Site is grouped by the geographical variation between samples from China, Denmark and the USA.

Three surgical methods—SG, LSG, and RYGB—all enhanced the α-diversity index of the gut microbiota, which was consistent with previous studies (Liu et al., 2017; Paganelli et al., 2019)(**Figures 1A, B**; **Supplementary Table S1**). Additionally, even within the same group (Healthy or OB) across different cohorts, significant differences were observed in alpha diversity (**Supplementary Figure S1**). To compare the effects of different surgical methods on gut microbiota diversity, for each cohort, we computed the ratio of the post-surgical α-diversity index increase relative to the OB group. The results showed that, in terms of species richness (ACE index), LSG > RYGB > SG. From the perspective of both species richness and evenness (Shannon index), RYGB > LSG > SG (**Supplementary Table S2**).

Principal Coordinates Analysis (PCoA) and PERMANOVA can be used to assess the impact of different studies and confounding factors on microbiota outcomes (Wang et al., 2020). The analysis revealed that cohort locations and surgical interventions significantly influence microbial community structure, with the impacts of obesity and surgical intervention being the most pronounced (indicated by higher R^2^ values). **Figure 1C** shows significant differences between the four cohorts (PRJEB12123, PRJEB12947, PRJNA597839, and PRJNA668357), with an PERMANOVA R^2^ value of 0.03539 and a p-value of 0.001, indicating significant grouping by cohort. **Figure 1D** illustrates the distribution of microbial communities across various health and treatment statuses, including healthy individuals and those undergoing LSG, RYGB, and SG. The analysis yielded a PERMANOVA R^2^ value of 0.06557 and a p-value of 0.001, indicating significant differences among these groups, with the impact of surgical treatment (a higher R^2^) being more pronounced than the influence of cohorts. To further validate the robustness of PERMANOVA results, we conducted variance partitioning analysis (VPA) (**Figure 1E**), which confirmed that the dominant effect of Treat is not influenced by the collinearity of project cohorts or country site, while the PERMANOVA R² of country site is inflated due to collinearity. Specifically, Treat exhibited an independent contribution of 6.75% (after excluding project cohorts and country site), whereas project cohorts contributed only 0.77% (after excluding country site and Treat), and country site showed no significant independent contribution (after excluding project cohorts and Treat), reinforcing that Treat is the primary variable with significant independent explanatory power. Overall, these results highlight the significant effects of cohort and surgical treatment on gut microbiota diversity.

### 3.2 Comparing differential analysis methods to identify gut significantly enriched microbes across cohorts

**Figure 2:**
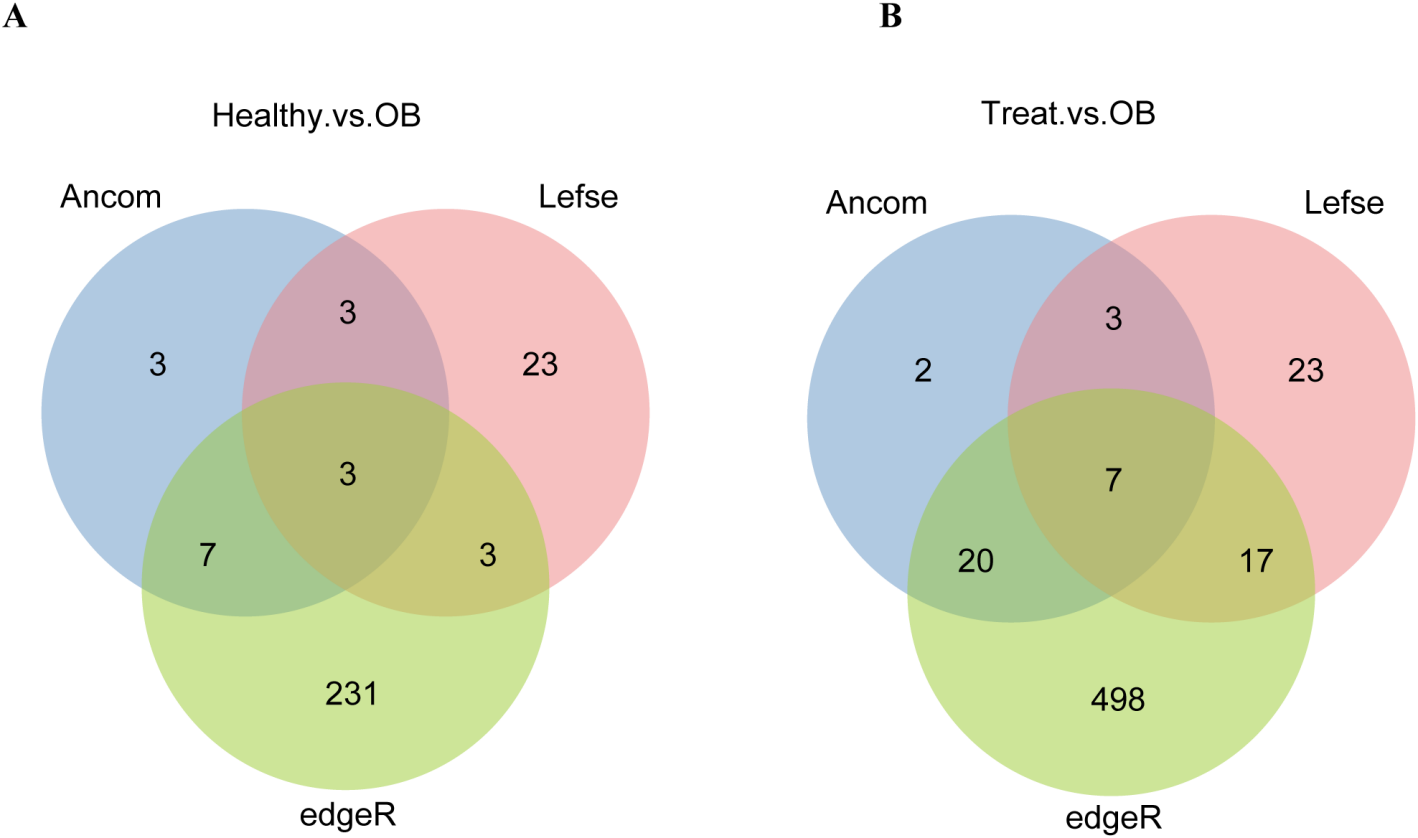
The Venn diagrams compare species markers identified by different methods (ANCOM, LEfSe, edgeR) for “Healthy vs. OB” **(A)** and “Treat vs. OB” **(B)** groups, highlighting unique and shared markers.

Due to the significant impact of different cohorts on gut microbiota, and to uncover significantly enriched microbes for subsequent MAGs analysis, we compared the results by three differential analysis methods for each cohort to identify differential species which enhances the accuracy of differential species analysis. For LEfSe differential analysis method: using LDA > 2, 51 differential species were identified between the OB and Treat groups, and 32 differential species were identified between the OB and Healthy groups. For ANCOM differential analysis method: using a detection threshold > 0.9, 32 differential species were identified between the OB and Treat groups, and 16 differential species were identified between the OB and Healthy groups. For edgeR differential analysis method: using FDR < 0.05 and fold-change > 5, 542 differential species were identified between the OB and Treat groups, and 244 differential species were identified between the OB and Healthy groups. The three differential analysis methods yielded varying results, with the number of identified markers ranked from highest to lowest as follows: edgeR, LEfSe, and ANCOM. However, there was relatively little overlap between the results of the three methods (**Figure 2**). Specifically, the three methods identified only 3 common markers in the “Healthy vs. OB” group and 7 common markers in the “Treat vs. OB” group, which is significantly fewer than the unique markers identified by each method. This highlights the necessity of integrating different cohorts and multiple differential analysis methods to comprehensively identify differential species.

### 3.3 Identified NonOB_Enrich and OB_Enrich microbes through consistent presence across cohorts and multi-methods

Theoretically, potential beneficial microbes are more likely to be enriched in healthy or treatment groups compared to the OB group, a strategy also utilized in a published study (Wu et al., 2024). More cohort data and difference methodological support could help minimize the likelihood of false-positive markers. In this context, the more diverse the differential analysis methods and the more supported cohorts, the more reliable the identified biomarkers will be. **Supplementary Table S3** lists species that are significantly enriched in the healthy group (16 species in total) and treatment group (11 species in total), focusing on those with the strongest support. For instance, *Coprobacter fastidiosus* and *Lachnospira eligens* were identified in two cohorts (PRJEB12123 and PRJNA597839) and supported by three differential analysis methods (edgeR, ANCOM, and LEfSe for the former; and ANCOM and LEfSe for the latter). Other species, such as *Bacteroides stercorisrooris*, *Bacteroides timonensis*, and *Brotilimocola acetigignens*, were identified in two cohorts and supported by edgeR analysis. In the treatment group, several species were identified across four cohorts, indicating strong support for their enrichment post-treatment. For example, *Bifidobacterium dentium*, *Raoultella planticola*, and *Streptococcus mutans* were consistently enriched across all four cohorts (PRJEB12123, PRJNA597839, PRJEB12947, and PRJNA668357), supported by edgeR analysis. Additionally, species such as *Veillonella atypica* and *Veillonella parvula* were identified in three cohorts and supported by three differential analysis methods (edgeR, ANCOM, and LEfSe). Similarly, we also analyzed the species enriched in OB group (**Supplementary Table S4**), including *Faecalimonas umbilicata*, *Fusobacterium varium*, *Acidaminococcus provencensis*, *Alkalihalobacillus bogoriensis*, *Collinsella tanakaei*, *Ellagibacter isourolithinifaciens*, *Enterococcus casseliflavus*, *Fusobacterium ulcerans*, *Limosilactobacillus mucosae*, *Peptacetobacter hiranonis*, *Slackia isoflavoniconvertens*, and *Turicibacter bilis*. Overall, 39 species biomarkers were identified, including 27 microbes enriched in the NonOB_Enrich group and 12 microbes enriched in the OB_Enrich group. These 39 identified species biomarkers could serve as potential beneficial or non-beneficial microbes for future wet-lab functional validation.

### 3.4 Functional insights from species-specific biomarker MAGs and differences in CAZY enzyme families

**Figure 3:**
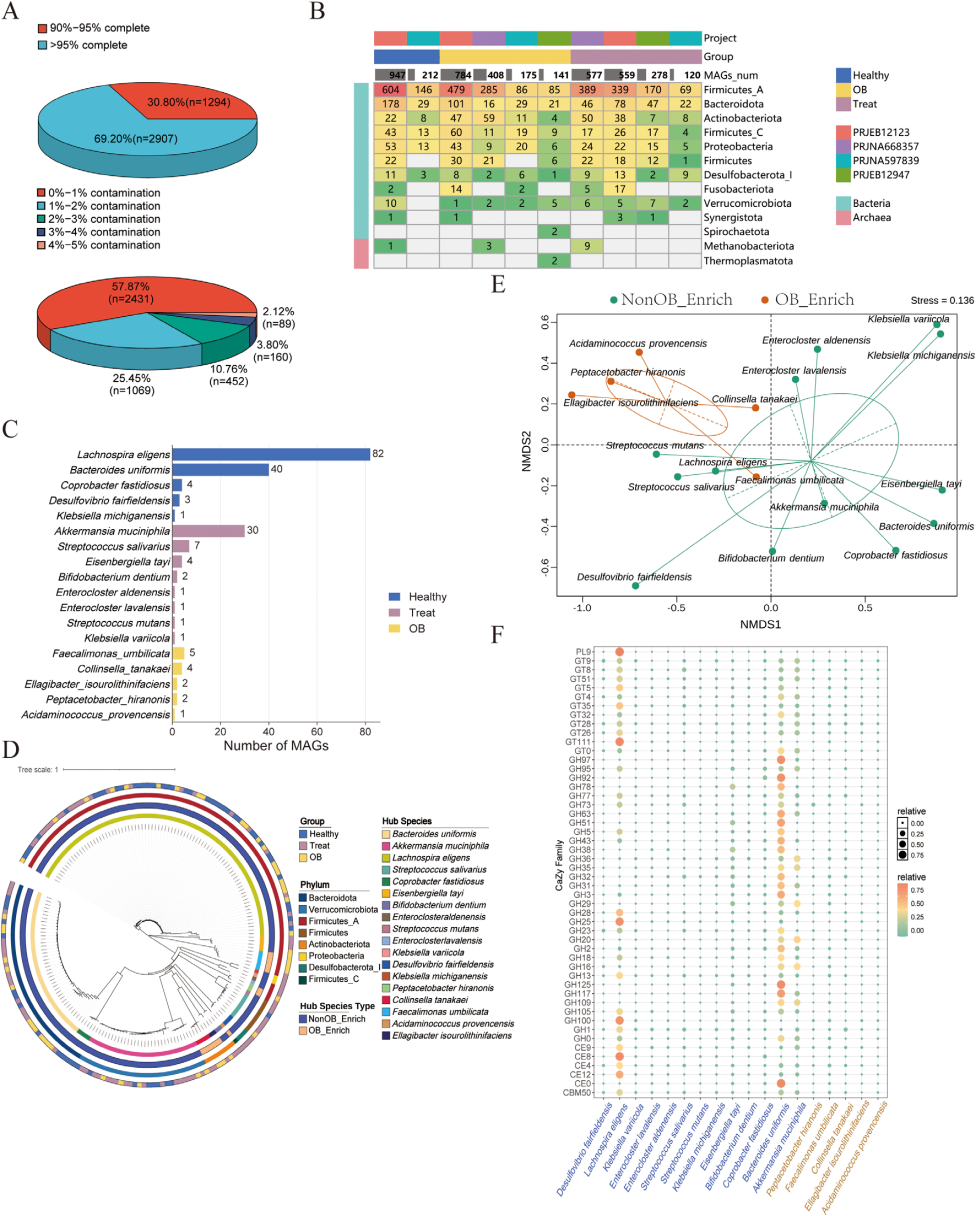
Comprehensive analysis of MAGs quality, distribution, and functional annotations. **(A)** Distribution of the 4,201 unique MAGs based on completeness or contamination levels. **(B)** The heatmap displays the distribution of MAGs across different corhots (PRJEB12123, PRJNA597839, PRJEB12947, PRJNA668357) and groups (Healthy, OB, Treat), organized by phylum. The color intensity and numerical values indicate the number of MAGs in each category. **(C)** Number of high-quality MAGs corresponding to potential species enriched in Healthy, OB and Treat group. **(D)** Phylogenetic tree of the MAGs. The rings from inner to outer represent species classifications, enriced-group, phylum and group affiliations (Healthy, Treat, OB). **(E)** NMDS analysis based on CAZy enzymes families relative abundance profile of MAGs in the NonOB_Enrich and OB_Enrich group. The clustering circles represent the 80% confidence ellipse based on s.class function in ade4 **(F)** Bubble chart showing the distribution of top 50 enzyme genes from MAGs annotated to CAZy enzyme families for MAGs in the NonOB_Enrich and OB_Enrich group.

To elucidate the genomic mechanisms underlying postoperative species abundance variations, we conducted a MAG construction analysis. We obtained 7,336 high-quality MAGs (completeness >90%, contamination <5%). After dRep-based deduplication, 4,201 unique MAGs were retained. We plotted the assembly completeness and contamination statistics for these MAGs. The quality statistics of the MAGs (**Figure 3A**) show that 69.2% of MAGs exceed 95% completeness, with the majority (57.87%) having contamination rates below 1%. The phylogenetic tree (**Supplementary Figure S2**) illustrates the diversity and distribution of MAGs across different groups, projects, and phyla. From the overall structure of the phylogenetic tree, MAGs from different phyla are classified into distinct branches based on their evolutionary relationships. The genomes are distributed across different research cohorts and groups, demonstrating overall high genomic diversity. According to **Figure 3B**, Firmicutes_A has the highest representation across all categories, particularly in the PRJEB12123 project and the Healthy group. Bacteroidota and Actinobacteriota also show significant presence, with notable counts in both the Healthy and Treat groups across multiple projects. Other phyla, such as Desulfobacterota_I, Fusobacteriota, and Verrucomicrobiota, have lower representation but are still present across different categories, indicating a diverse genomic composition.

Based on the significantly enriched microbe lists in **Supplementary Tables S3**, **S4**, the number of MAGs identified through binning for each bacterium was summarized (**Supplementary Table S5**; **Figure 3C**). The analysis identified 185 gut microbiota MAGs (171 MAGs in the NonOB_Enrich group and 14 MAGs in the OB_Enrich group). These MAGs include five core species in the healthy group: *Lachnospira eligens*, *Bacteroides uniformis*, *Coprobacter fastidiosus*, *Desulfovibrio fairfieldensis*, and *Klebsiella michiganensis*. In the treatment group, eight core species were identified: *Akkermansia muciniphila*, *Streptococcus salivarius*, *Eisenbergiella tayi*, *Bifidobacterium dentium*, *Enterocloster aldenensis*, *Enterocloster lavalensis*, *Streptococcus mutans*, and *Klebsiella variicola*. Notably, *Lachnospira eligens* exhibited the highest number of MAGs in the healthy group, followed by *Bacteroides uniformis* and *Coprobacter fastidiosus*. In the treatment group, *Akkermansia muciniphila* had the highest number of MAGs. Additionally, five core species were identified, including *Faecalimonas umbilicata*, *Collinsella tanakaei*, *Ellagibacter isourolithinifaciens*, *Peptacetobacter hiranonis*, and *Acidaminococcus provencensis*. The phylogenetic tree results (**Figure 3D**) show that *Bacteroides uniformis*, *Coprobacter fastidiosus*, and *Akkermansia muciniphila* in the NonOB_Enrich group, cluster into one branch, while *Collinsella tanakaei* and *Ellagibacter isourolithinifaciens* in the OB_Enrich group, form a separate branch. These species may exhibit certain similarities in their biological functions. We further conducted a literature review to validate the aforementioned microbes as potential beneficial or non-beneficial ones (**Supplementary Table S6**).

A key characteristic of obesity-related microorganisms is their ability to metabolize carbohydrates, converting specific sugars into propionic acid, lactic acid, or acetic acid. This metabolic activity plays a significant role in modulating the host’s response to a carbohydrate-rich diet, which is particularly relevant in the context of obesity development (Derrien and van Hylckama Vlieg, 2015; Le Barz et al., 2015; Schwiertz et al., 2010). NMDS based on CAZY enzymes count of MAGs (**Figure 3E**) showed a significant separation between microbes in the NonOB_Enrich and OB_Enrich group, indicating distinct carbohydrate degradation and utilization profiles. The bubble plot (**Figure 3F**) further shows the top 50 enzyme families by relative gene abundance across various species. The analysis reveals that key enzyme families, including Carbohydrate-Binding Modules (CBM), Carbohydrate Esterases (CE), Glycoside Hydrolases (GH), GlycosylTransferases (GT), and Polysaccharide Lyases (PL), have a higher relative proportion of metabolic enzyme genes detected in MAGs of the NonOB_Enrich group, such as *Bacteroides uniformis*, *Lachnospira eligens*, *Akkermansia muciniphila*, and *Eisenbergiella tayi*, compared to the MAGs in OB_Enrich group, implying that they have a stronger ability to metabolize and utilize a variety of complex carbohydrates.

### 3.5 Microbes enriched in the NonOB_Enrich group, show specific genetic adaptations enhancing essential metabolic functions for gut health

**Figure 4:**
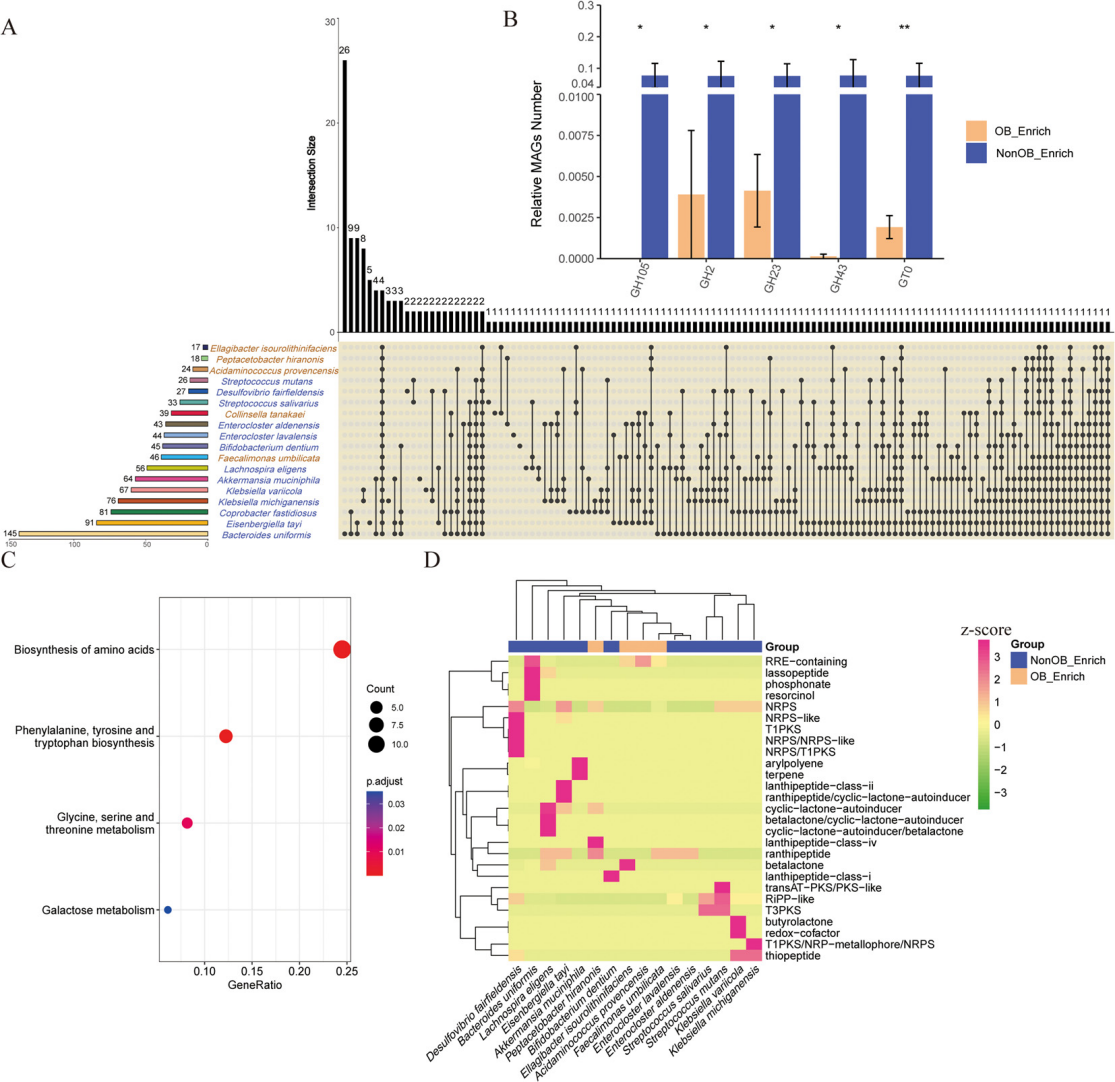
Statistical and enrichment analysis of functional annotation results MAGs in the NonOB_Enrich and OB_Enrich group. **(A)** Shared and unique CAZy enzymes detected in microorganisms. Vertical bars (left): Represent the total number of CAZy enzymes in each microbe individually. Horizontal bars (above): Show the size of intersections, indicating the number of enzymes shared among microbes or unique to each group. Dots and connecting lines: Illustrate the specific combinations of sets being compared. **(B)** Significantly different enzyme families between the NonOB_Enrich group and the OB_Enrich group. **(C)** KEGG enrichment analysis based on the significantly different KEGG KOs. **(D)** Cluster analysis of secondary metabolite prediction based on MAGs. Normalization of the average number of secondary metabolites detected in each species’ MAG using Z-scores. * indicates statistical significance at P < 0.05, while ** denotes high statistical significance at P < 0.01.

We compared the shared and unique CAZy enzymes detected in microbes between the NonOB_Enrich and OB_Enrich group (**Figure 4A**; **Supplementary Table S7**). The results indicate that microbes in NonOB_Enrich group exhibit a significantly higher number of CAZy enzyme types compared to the OB_Enrich group microbes. The top three enzymes are associated with *Bacteroides uniformis*, *Eisenbergiella tayi*, and *Coprobacter fastidiosus*, while the bottom three are linked to *Ellagibacter isourolithinifaciens*, *Peptacetobacter hiranonis*, and *Acidaminococcus provencensis*. This suggests a greater diversity in the carbohydrate-active enzyme profiles of microbes in the NonOB_Enrich group. Additionally, *Bacteroides uniformis* possesses the highest number of unique enzymes, followed by a small number of enzyme families shared among some microbes. The relative proportion of genes from GH105, GH2, GH23, GH43, and GT0 gene families is significantly higher in microbes of the NonOB_Enrich group compared to the microbes in OB_Enrich group (**Figure 4B**).

Based on the 74 significantly different KOs (**Supplementary Table S8**), these KOs were significantly enriched in four pathways: “amino acid biosynthesis,” “glycine, serine, and threonine metabolism,” “phenylalanine, tyrosine, and tryptophan biosynthesis,” and “galactose metabolism” (**Figure 4C**). The result in **Figure 4D** shows that species enriched in the NonOB_Enrich group produced more secondary metabolites compared to those in the OB_Enrich group. Furthermore, the predominant secondary metabolites produced by different species varied. For instance, *Akkermansia muciniphila* produced more terpenes, *Bacteroides uniformis* produced more RRE-containing compounds, and *Lachnospira eligens* produced more cyclic-lactone autoinducers. Additionally, the clustering results for the NonOB_Enrich and OB_Enrich groups displayed a clear separation trend, with the exception of *Bifidobacterium dentium*. In healthy human gut microbiota, secondary metabolite genes, such as those involved in NRPS, terpenes, and RiPPs, helped maintain microbial diversity and inhibited excessive pathogen growth. In contrast, in obese individuals, secondary metabolite gene diversity of MAGs was reduced, and the metabolite profile was altered, potentially contributing to chronic low-grade inflammation and metabolic disorders.

### 3.6 Comparison of fatty acid biosynthesis genes and PTR across obesity-associated and non-obesity-associated MAGs

**Figure 5:**
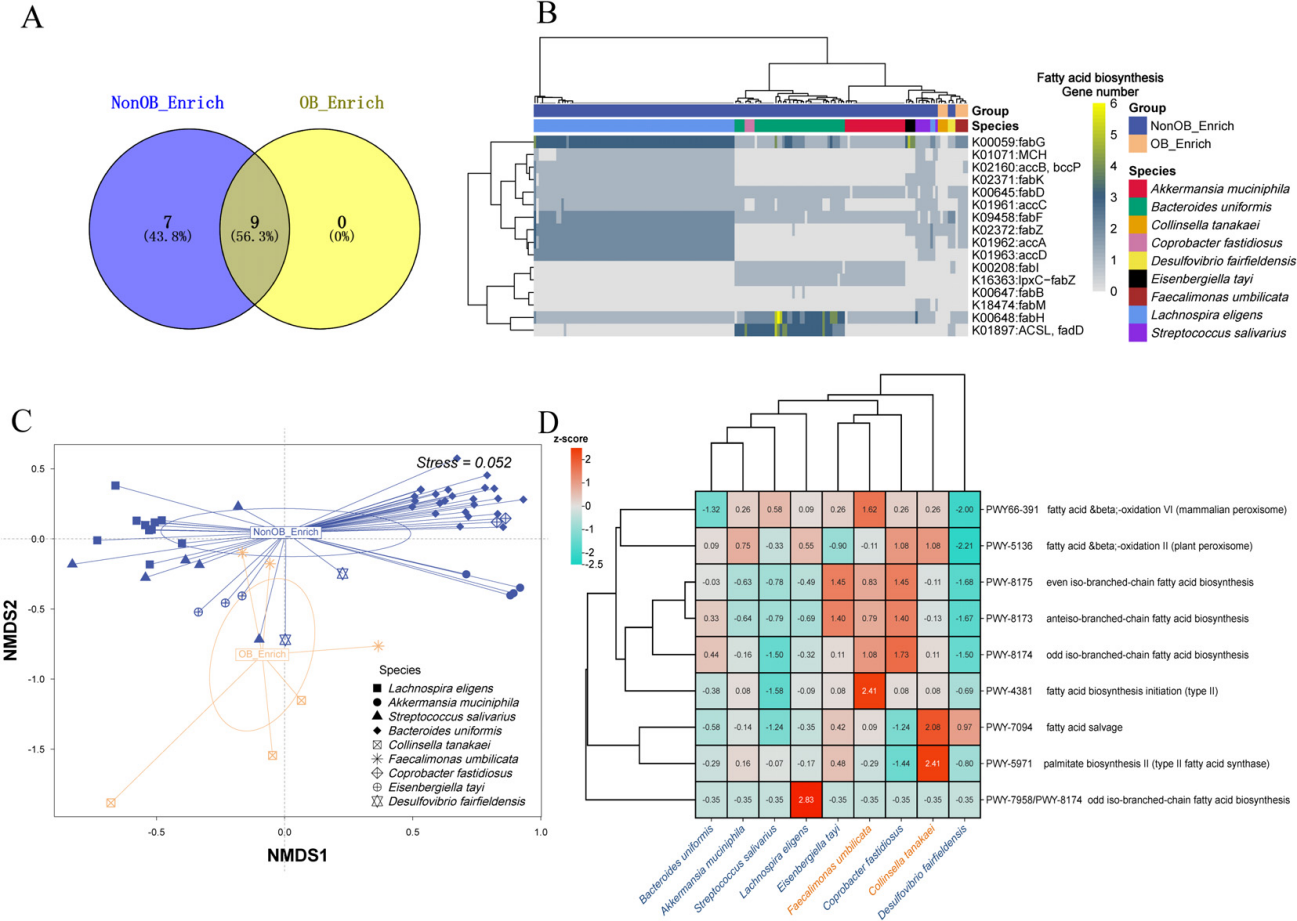
Analysis of fatty acid biosynthesis genes and reactions distribution. **(A)** Venn diagram showing the distribution of fatty acid biosynthesis genes in the “NonOB_Enrich” and “OB_Enrich” groups. The numbers indicate the number of unique and shared genes between the two groups. **(B)** The heatmap illustrates the presence of various fatty acid biosynthesis genes across different species. Clustering was performed on both rows and columns using the ward.D2 method, with normalization applied by row **(C)** Non-metric multidimensional scaling (NMDS) plot showing the distribution of species in the OB_Enrich and NonOB_Enrich groups. The clustering circles represent the 80% confidence ellipse based on s.class function in ade4. **(D)** a clustering heatmap analysis comparing the average number of reactions predicted for each MAG in the fatty acid biosynthesis pathway across species containing three or more MAGs based on gapseq.

Microbes that activate the fatty acid biosynthesis pathway can induce body weight gain (Henneke et al., 2022). We compared the presence of fatty acid biosynthesis genes across species in the OB_Enrich and NonOB_Enrich (each species containing at least three MAGs included in the analysis). The Venn analysis results in **Figure 5A** show that more genes were detected in the NonOB_Enrich group, with seven unique genes, while both groups shared nine detected genes. This is consistent with a previous report, where the activity of genes involved in fatty acid metabolism is relatively low, particularly those related to enzymes in the tricarboxylic acid (TCA) cycle, with their function being significantly reduced (Liu et al., 2017). **Figure 5B** shows that the MAGs of different species clustered together, indicating a preference for the fatty acid biosynthesis genes carried by each species. The beneficial gut bacterium *Lachnospira eligens* carried the most diverse set of genes, including *fabG, MCH, accB/bccP, fabK, fabD, accC, fabF, fabZ, accA*, and *accD*. *Bacteroides uniformis* carried *fabG, fabD, fabF, fabI, IpxC-fabZ, fabH*, and *ACSL/fadD*. *Akkermansia muciniphila* carried *fabG, fabD, fabF, fabI, IpxC-fabZ*, and *fabH*. The species enriched in OB_Enrich, *Collinsella tanakaei*, carried the fewest genes, only including *fabF* and *fabZ*. The result in **Figure 5C** further shows a tendency for separation between the OB_Enrich MAGs and NonOB_Enrich MAGs, indicating a distinct difference in the detection profiles of fatty acid biosynthesis genes between the two groups. Overall, NonOB_Enrich MAGs exhibit higher gene diversity, which may contribute to SCFA production, promote fatty acid oxidation rather than storage, and reduce lipid accumulation, thereby regulating and improving host obesity (Den Besten et al., 2013; Gurung et al., 2020). This may explain why obesity is significantly associated with a reduction in SCFA concentration (Ecklu-Mensah et al., 2023).

We performed a clustering heatmap analysis (**Figure 5D**) to compare the average number of key reactions (z-score normalized) related to the fatty acid biosynthesis pathway, which were predicted from the genome fasta sequences of species in the obesity-associated microbiome group (OB_Enrich) and the non-obesity-associated group (NonOB_Enrich). The analysis revealed that beneficial gut microbes related to short-chain fatty acid production, such as *Bacteroides uniformis, Akkermansia muciniphila, Streptococcus salivarius*, and *Lachnospira eligens*, clustered into one subgroup. This indicates that these four species exhibit more similar fatty acid biosynthesis pathway reactions, suggesting potential synergistic effects. However, *Coprobacter fastidiosus*, *Eisenbergiella tayi*, and *Desulfovibrio fairfieldensis*, which were enriched in the NonOB_Enrich group, did not cluster with the other four species mentioned above, indicating distinct metabolic regulation mechanism. The enrichment of these three species in NonOB_Enrich may not directly regulate body weight through fatty acid biosynthesis, but rather exert their effects through other mechanisms, such as improving inflammatory responses.

Microbial growth rate, as reflected by PTR, was linked to disease status and largely independent of relative abundances capturing a unique biological variation that complements relative abundance data (Joseph et al., 2022a). Based on PTR analysis (**Supplementary Table S9**), we discovered that microbes in the NonOB_Enrich group and OB_Enrich group mainly clustered into two distinct branches (**Supplementary Figure S3A**). The growth rates of the same species exhibited varying distribution patterns across different dimensions, such as geographic regions, study cohorts, and participant groups. Further analysis using differential boxplots revealed that in the PRJNA668357 cohort, *Akkermansia muciniphila* (**Supplementary Figure S3B**) and *Bacteroides uniformis* (**Supplementary Figure S3C**) had significantly higher PTRs in the RYGB group compared to the OB group. Considering the longitudinal data across different postoperative time points, *Akkermansia muciniphila* showed a significant increasing trend in PTR from the 1st to the 6th month after RYGB surgery (**Supplementary Figure S3D**), although this trend became non-significant by the 12th month (*p-*value = 0.075). For *Bacteroides uniformis*, there was a significant increase in PTR from the 1st to the 12th month post-surgery (**Supplementary Figure S3E**), with the increase at the 6th month being less pronounced (*p* value = 0.18). No statistically significant differences were observed in the PTRs of other microbes after surgery. This further indicates that *Akkermansia muciniphila* and *Bacteroides uniformis*, play a more crucial role in improving obesity, with different microbes playing crucial roles at various stages of recovery.

## 4 Discussion

**Figure 6:**
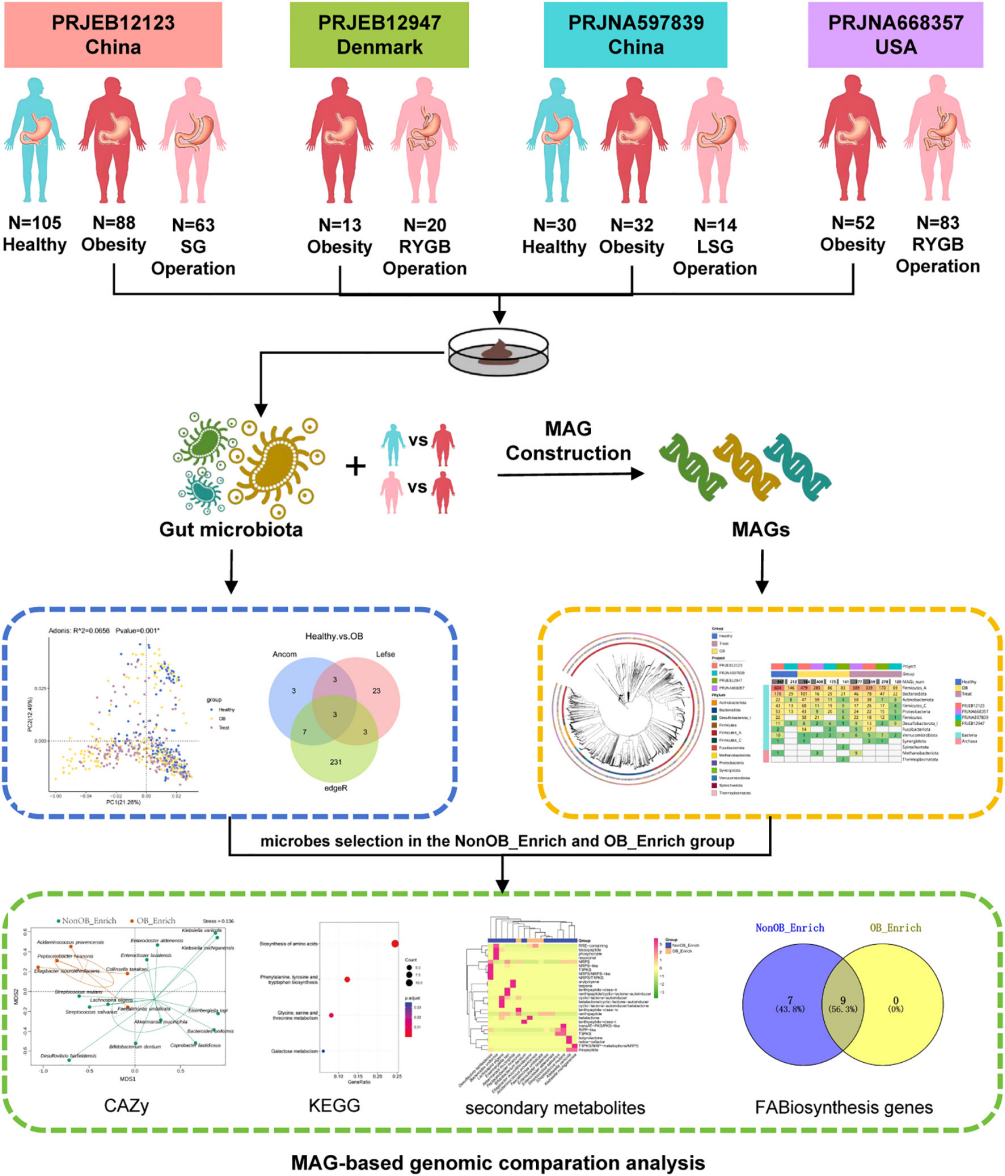
The diagram outlines a gut microbiota study comparison of MAGs in the NonOB_Enrich and OB_Enrich group across different cohorts, including various obesity and bariatric surgery groups. It begins with sample collection and MAG construction, followed by the exploration of microbial species and genomic composition. The study identified significantly enriched microbial biomarkers, followed by a MAGs-based genomic comparison to explore their metabolic functions and contributions to gut health, including KEGG KO comparison, CAZy enzyme comparison, secondary metabolites, key fatty acid biosynthesis (FABiosynthesis) genes comparison. This research offers valuable insights into the discovery of weight loss microbes and their underlying mechanisms.

Bariatric surgeries, effective for severe obesity, result in significant changes to gut microbiota, highlighting the importance of identifying weight loss-related microbes and understanding their mechanisms to advance both human health and the health industry. As the first metagenome-based comprehensive analysis integrating multiple studies on bariatric surgery and gut microbiota (**Figure 6**), this study’s methodology involves 4 cohorts and surgical interventions (Sleeve Gastrectomy, Laparoscopic Sleeve Gastrectomy, Roux-en-Y Gastric Bypass) in 3 countries (China, Denmark, USA) using a standardized bioinformatics analysis pipeline. Through cross-cohort integration of multiple differential analysis methods, the study identified significantly enriched microbial biomarkers, followed by a MAGs-based genomic comparison to explore their metabolic functions and contributions to gut health, including KEGG KO comparison, CAZy enzyme comparison, secondary metabolites, key fatty acid biosynthesis genes and reactions comparison. This research provides valuable insights into the discovery of weight loss microbes and their underlying mechanisms.

SG, LSG, and RYGB are common bariatric procedures used to treat obesity. However, RYGB is generally considered more effective in achieving long-term weight loss and remission of metabolic diseases (Mingrone et al., 2012; Schauer et al., 2017). The results of this study indicate that RYGB has a superior impact on restoring gut microbiota α-diversity (shannon index) compared to the other two procedures, which may contribute to more effective treatment of obesity. RYGB surgery reduces the size of the stomach and bypasses a portion of the small intestine, thereby significantly altering the digestive pathway. As a result, food no longer passes through the duodenum and proximal jejunum, which changes the absorption of nutrients and, in turn, impacts the microbial environment and composition of the gut microbiota. Additionally, changes in bile acid metabolism following RYGB may promote the growth of beneficial bacterial species, thereby contributing to the restoration of microbial α-diversity (Furet et al., 2010).

Identifying differentially abundant microbes is a key objective in microbiome research, with various methods often used interchangeably in the literature (Nearing et al., 2022). However, studies frequently report inconsistent results regarding the microbial effects of specific bacteria (D’Elios et al., 2020; Suez et al., 2019), and a method that performs well in one study may be absent from another (Nearing et al., 2022). In our study, based on species-level relative abundance, three methods (edgeR, ANCOM, and LEfSe) identified only 3 shared markers in the “Healthy vs. OB” group and 7 in the “Treat vs. OB” group, which is substantially fewer than the number of unique markers identified by each method. These findings underscore the need for integrating diverse cohorts and utilizing multiple differential analysis methods to achieve a more comprehensive identification of differential species.

A key characteristic of beneficial microorganisms is their ability to metabolize carbohydrates, converting specific sugars into propionic, lactic, or acetic acid. Microbes in the NonOB_Enrich group exhibit a significantly higher number of CAZy enzyme types compared to the OB_Enrich group, with gene families such as GH105, GH2, GH23, GH43, and GT0 being significantly more abundant. This is primarily because beneficial microbes typically inhabit the host’s gut (e.g., humans), forming a symbiotic relationship closely tied to the host’s nutritional needs (Lee and Mazmanian, 2010). Gut beneficial microbes help the host digest food, break down complex carbohydrates, synthesize essential amino acids, and maintain gut microecological balance. These functions require specific metabolic capabilities, such as the breakdown of carbohydrates by enzymes like alpha-galactosidase and the synthesis of tryptophan by tryptophan synthase. For example, *Coprobacter fastidiosus* expresses a significant number of glycoside hydrolase (GH)-encoding genes and possesses the highest diversity of GH families, thereby promoting the host’s ability to digest an appropriate high-fiber diet (Henneke et al., 2022).

In the analysis of fatty acid biosynthesis, *Coprobacter fastidiosus, Eisenbergiella tayi*, and *Desulfovibrio fairfieldensis*, which were enriched in the NonOB_Enrich group, did not cluster with the other species. A possible explanation for this could be related to the unique metabolic pathways of *Coprobacter fastidiosus*. Propionate, acetate, and succinate are generated through the Wood-Werkman cycle and a partial tricarboxylic acid (TCA) cycle. Genomic analysis of the core metabolism of *C. fastidiosus* suggests the presence of a Wood-Werkman cycle and a partial TCA cycle, which lacks succinyl-CoA hydrolase, similar to the pathway described for Propionibacterium freudenreichii. Additionally, *C. fastidiosus* possesses a respiratory chain that can be utilized in propionic acid production. Instead of following the classic fatty acid biosynthesis pathway, acetyl-CoA in *C. fastidiosus* is likely synthesized via pyruvate-flavodoxin oxidoreductase (gene NSB1T_03985) (Chen et al., 2024). In longevity populations, *D. fairfieldensis* is more abundant and contributes to the biosynthesis of menaquinone (vitamin K2), which may help prevent age-related diseases, such as osteoporosis-induced fractures. *E. tayi* in the gut plays a key role through enzymes encoded in its genome, including 1.17.1.8: 4-hydroxy-tetrahydrodipicolinate reductase (EC 1.17.1.8), 4.3.3.7: 4-hydroxy-tetrahydrodipicolinate synthase (EC 4.3.3.7), and 2.7.2.4: Aspartate kinase (EC 2.7.2.4), which are important for distinguishing the transcriptional states of keystone taxa (Bauchinger et al., 2024). Moreover, studies on the gut microbiome of longevity populations have found that *E. tayi* is more abundant in these groups. The bacterial protein N-glycosylation involving *E. tayi* may play a significant role in regulating physiological and pathological processes during aging (Chen et al., 2024). The enrichment of these three species in the NonOB_Enrich group may not directly influence body weight through fatty acid biosynthesis, but rather may exert effects via other mechanisms.

PTRs have the potential to be a valuable tool for investigating microbiome dynamics (Joseph et al., 2022b). From the perspective of PTRs based on the constructed MAGs, we found that in the USA cohort (PRJNA668357), *Akkermansia muciniphila* and *Bacteroides uniformis* exhibited significantly higher PTRs in the RYGB-treated group compared to the obese (OB) group, suggesting enhanced growth post-surgery. The longitudinal increase in PTRs for these species across different postoperative time points further highlights their potential roles in recovery and weight management. These PTRs increase emphasize the importance of specific biomarkers, particularly *Akkermansia muciniphila* and *Bacteroides uniformis*. Known for its ability to improve gut barrier integrity and metabolic health, *Akkermansia muciniphila* has been shown to reduce body fat, enhance insulin sensitivity, and decrease inflammation, making it a promising candidate for obesity treatment (Xu et al., 2020). Similarly, *Bacteroides uniformis* may support weight loss by improving metabolic functions and modulating gut microbiota, especially in obese individuals with intestinal dysbiosis. This beneficial effect has been observed in animal models, demonstrating potential for future applications in weight management (Gomez Del Pulgar et al., 2020; Van Hul and Cani, 2023). While PTR analysis provides insights into microbial replication dynamics, its interpretation should be tempered by methodological constraints. The accuracy of PTR estimates inherently depends on the completeness of MAG reconstruction and sequencing depth, as fragmented assemblies or low-coverage genomes may introduce biases in peak-to-trough ratio calculations. Therefore, this approach is primarily applicable for comparative analyses of high-quality MAGs, while its utility as an absolute measure of metabolic activity across heterogeneous microbial communities may be limited.

However, this study has three limitations. Firstly, it relies solely on metagenomic data, lacking comprehensive clinical metrics for deeper correlation analysis. Secondly, the use of Kraken for species identification is restricted to known microbial databases, leaving potential unknown microbes uninvestigated. Thirdly, the study is limited to metagenomic analysis without integrating multi-omics data or conducting further validation experiments on the identified microbe biomarkers.

This study contributes to the understanding of obesity treatment by identifying microbial species associated with weight loss, through cross-cohort integration and multi-method analysis. It emphasizes the importance of gut microbiota restoration and microbial diversity, offering valuable insights for potential therapeutic applications.

## Data Availability

The datasets presented in this study can be found in online repositories. The names of the repository/repositories and accession number(s) can be found in the article/**Supplementary Material**.
