# Supplementary material for: MAGs-based genomic comparison of gut significantly enriched microbes in obese individuals pre- and post-bariatric surgery across diverse locations
Source: Front Cell Infect Microbiol. 2025 Mar 18;15:1485048. doi: 10.3389/fcimb.2025.1485048 (PMC11958714; doi:10.3389/fcimb.2025.1485048)
Supplement: Supplementary file 1 [file DataSheet1.pdf]

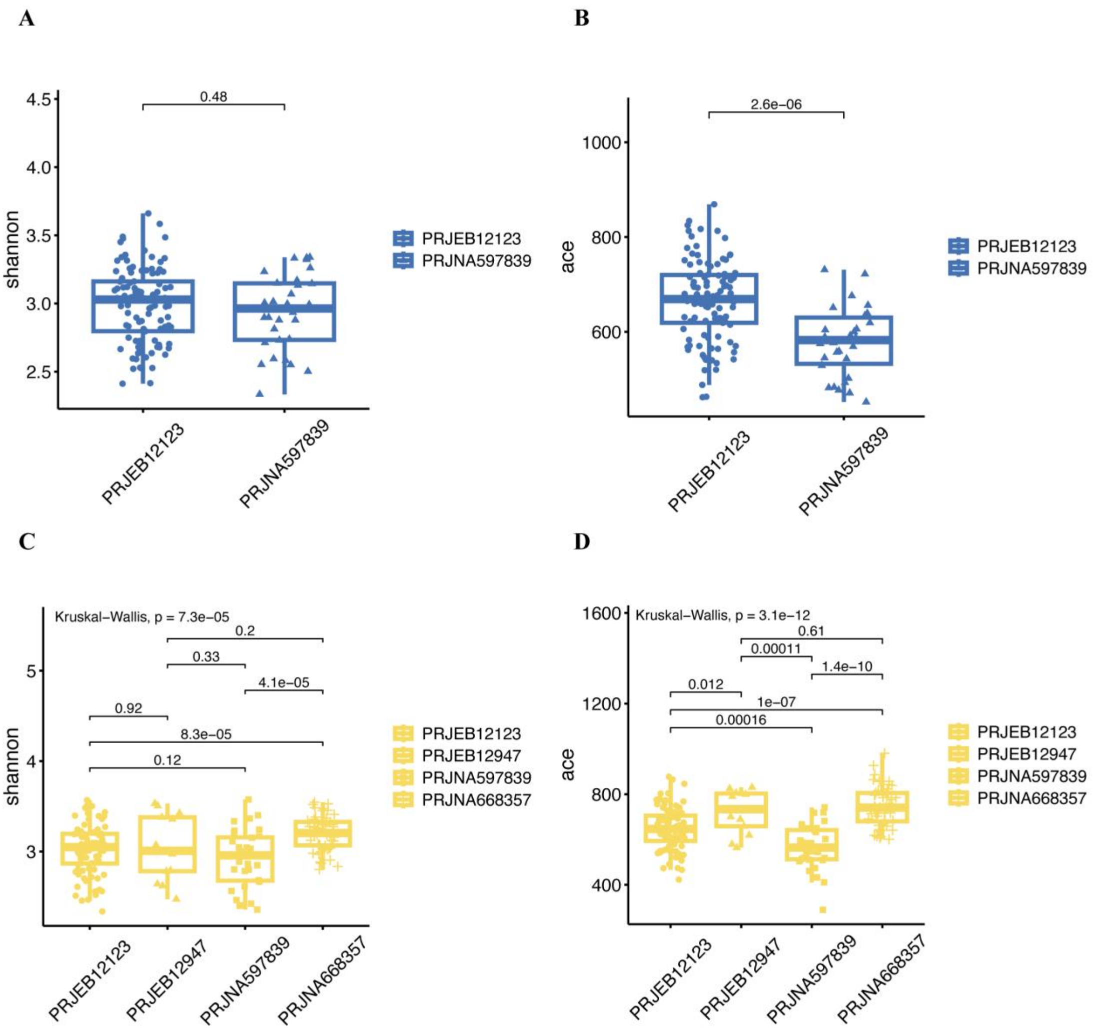

Figure S1. Comparison of diversity indices between different cohorts within the same group. **(A)**shannon comparation of Healthy group. **(B)**ace comparation of Healthy group. **(C)** shannon comparation of Obesity group. **(D)**ace comparation of Obesity group.

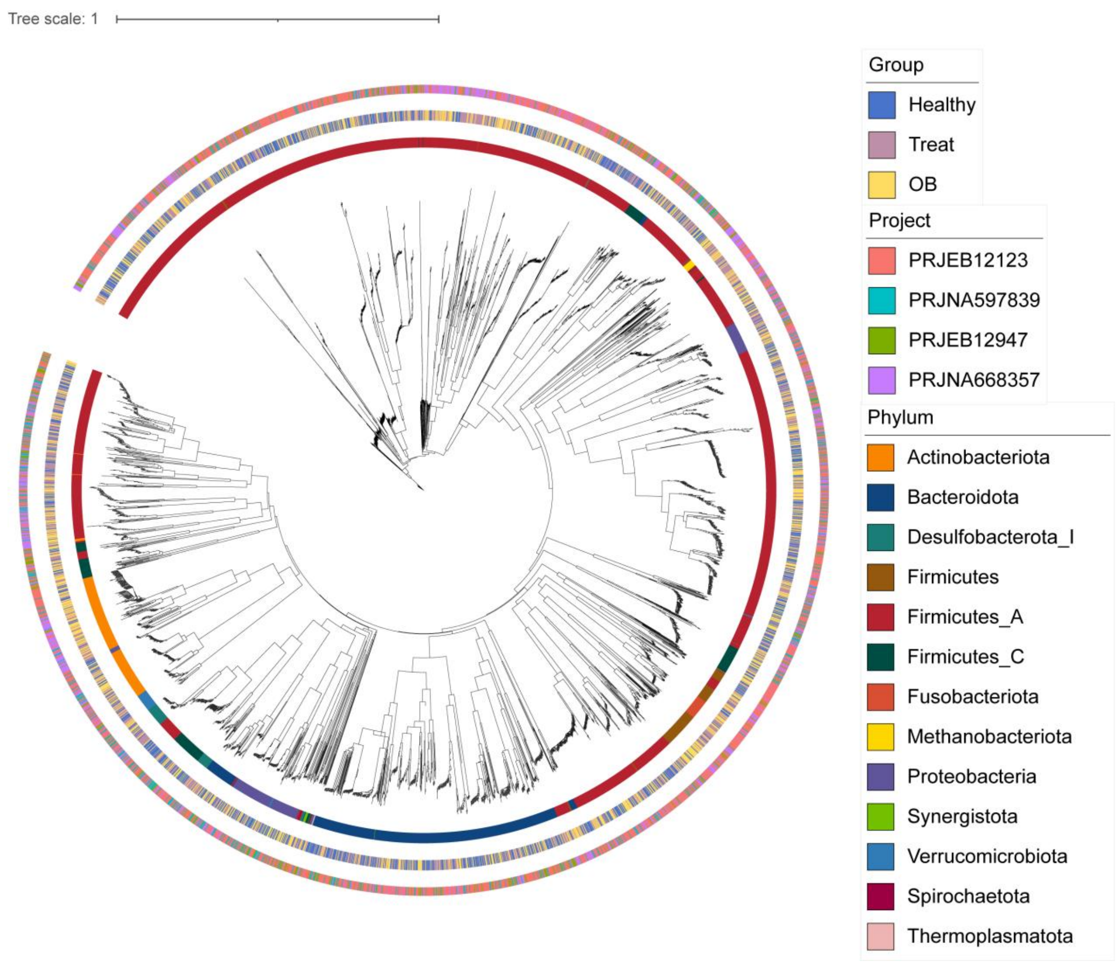

Figure S2. Phylogenetic tree of 4,201 MAGs. The rings from inner to outer represent phylum classifications (e.g., Actinobacteriota, Bacteroidota, Firmicutes), group affiliations (Healthy, Treat, OB), and project sources (PRJEB12123, PRJNA597839, PRJEB12947, PRJNA668357).

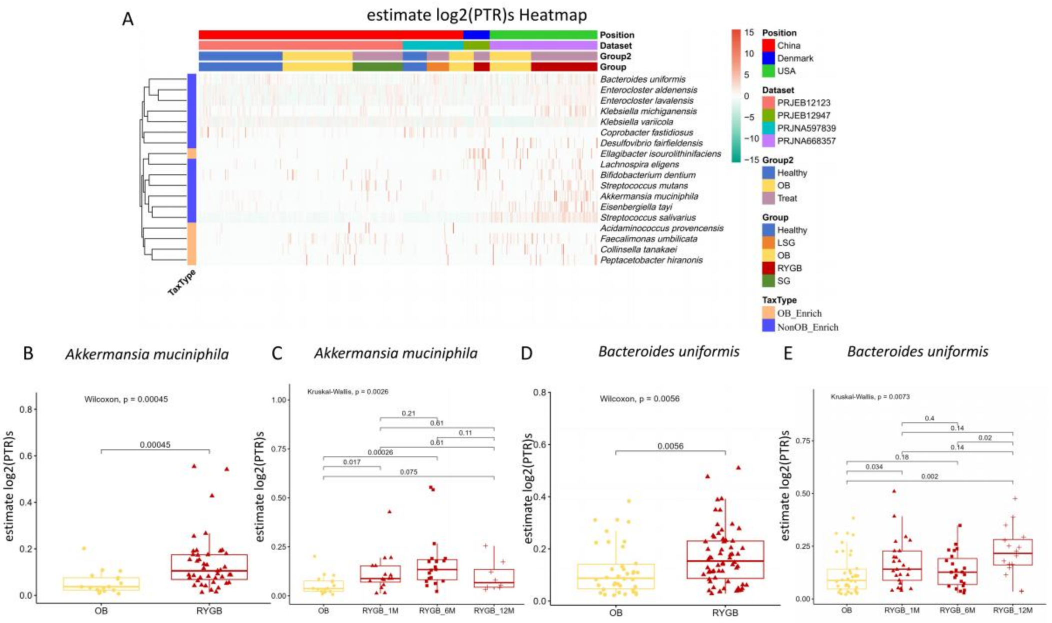

Figure S3. PTR comparison of MAGs in the NonOB_Enrich and OB_Enrich group. **(A)** PTR heatmap of microbes across different cohorts, groups, and countries. **(B)** PTR comparison of *Akkermansia muciniphila* between the OB and RYGB groups in the PRJNA668357 cohort. **(C)** PTR comparison of *Akkermansia muciniphila* among the OB and RYGB treatment groups at different stages in the PRJNA668357 cohort. **(D)** PTR comparison of *Bacteroides uniformis* between the OB and RYGB groups. **(E)** PTR comparison of *Bacteroides uniformis* among the OB and RYGB treatment groups at different stages in the PRJNA668357 cohort.
